# A highly selective and sensitive “on–off” fluorescent probe for detecting cadmium ions and l-cysteine based on nitrogen and boron co-doped carbon quantum dots†

**DOI:** 10.1039/d1ra08219a

**Published:** 2022-03-15

**Authors:** Zhihong Yan, Wei Yao, Kang Mai, Jiaqi Huang, Yating Wan, Liu Huang, Bo Cai, Yi Liu

**Affiliations:** College of Pharmacy, Guangdong Pharmaceutical University Guangzhou 510000 China Liuyi915@126.com; School of Pharmaceutical and Chemical Engineering, Guangdong Pharmaceutical University Zhongshan 528400 China; Zhongshan Carefor Daily Necessities Ltd Zhongshan 528400 China; Guangzhou OPSEVE Cosmetics Co. Ltd Guangzhou 510000 China

## Abstract

Cadmium ions (Cd^2+^) have caused relatively serious pollution, threatening human health and ecosystems. l-Cysteine (l-Cys) is an essential amino acid in living organisms and concentration of l-Cys is closely related to some human diseases. In this work, we first introduced 2-amino-3-hydroxypyridine and sodium borohydride as the nitrogen source and boron source to fabricate boron and nitrogen co-doped carbon quantum dots (N,B-CQDs) with high fluorescence quantum yield (21.2%), which were synthesized through a simple, low-consumption and pollution-free one-pot hydrothermal method. The obtained N,B-CQDs are able to detect Cd^2+^ rapidly and sensitively through fluorescence enhancement, which may be ascribed to chelation enhanced fluorescence that is induced by the formation of the N,B-CQDs/Cd^2+^ complex. Simultaneously, N,B-CQDs can be used to detect l-cysteine because significant fluorescence quenching was observed when l-Cys was added into the N,B-CQDs/Cd^2+^ system. In the two fluorescence “turn-on” and “turn-off” processes, this fluorescent probe obtained a good linear relationship over Cd^2+^ concentration ranging from 2.5 µM to 22.5 µM with a detection limit of 0.45 µM, while the concentration of l-cysteine showed a linear relationship in the range of 2.5–17.5 µM with a detection limit of 0.28 µM. The sensor has been successfully used to detect Cd^2+^ and l-cysteine in real samples with satisfying results.

## Abstract 2

Schematic presentation of the synthesis of N,B-CQDs and their application as an “on–off” fluorescent probe for Cd^2+^ and l-Cys detection.

## Graphical abstract

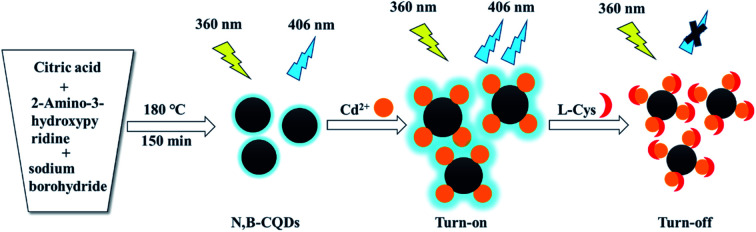

## 1. Introduction

It is well known that various heavy metal ions have caused relatively serious pollution, threatening human health and ecosystems. Among those various heavy metal ions, cadmium ions (Cd^2+^) in particular have been proven to be highly toxic and can cause human health problems.^1–4^ Continuous exposure to even a small amount of Cd^2+^ through ingestion of contaminated food or water can cause severe damage to the lung, kidney, bone and nervous system, and even lead to some cancers.^5–7^ Common detection methods for Cd^2+^ ions include atomic absorption spectrometry (AAS),^8^ inductively coupled plasma mass spectrometry (ICP-MS)^9^ and spectrophotometry.^10^ Although these methods have high sensitivity and multiple detection capabilities, their application of detection in many practical cases is limited by the complex sample pre-preparation process, high cost and susceptibility to interference.^11^l-cysteine (l-Cys), an essential amino acid found in living organisms, plays an indispensable role in cell reduction process and phospholipid metabolism in liver, and has pharmacological effects of protecting liver cells from damage, promoting liver function recovery and vitality.^12^ Therefore, it makes a lot of sense to develop simple methods to detect Cd^2+^ sensitively and selectively for human health and environmental protection.

In recent years, fluorescence method has become a popular detection method due to its advantages of simplicity, economy, high sensitivity and fast response speed. A variety of fluorescent probes have been researched, such as organic fluorophore molecules,^13^ metal nanoparticles^14,15^ and semiconductor quantum dots (QDs).^16^ However, the majority of the probes described above are usually either toxic or poor selective.

After more than a decade of development, carbon quantum dots (CQDs) have become a quite promising fluorescent probe with many unique advantages over traditional organic fluorophore molecules and semiconductor quantum dots, including excellent optical properties, favourable water solubility, low toxicity, excellent biocompatibility, and excellent sensitivity and selectivity.^17,18^ In addition, carbon quantum points have been widely used in the fields of biological imaging,^19,20^ catalysis^21^ and sensors.^22^ In the above applications, it has been demonstrated that not merely surface functionalization/passivation, but also heteroatomic doping can improve the performance of CQDs. Boron (B), nitrogen (N), sulfur (S) and phosphorus (P) are the most common heteroatomic doping in carbon-based materials. The adjacent elements boron and nitrogen of carbon (C) in the periodic table have atomic radii similar to C, which makes it possible to effectively alter electronic characteristics and modulate physicochemical properties of carbon-based materials after doping. There are some previous researches have been reported to synthesize B and N co-doped carbon quantum dots based materials.^23–26^ However, the strategy for innovative and brief ways to make new B and N co-doped CQDs remains challenging.

**Scheme 1:**
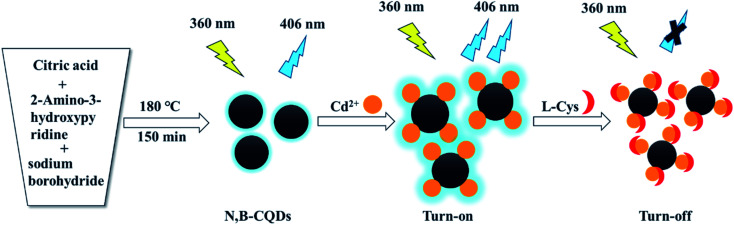
Schematic presentation of the synthesis of N,B-CQDs and their application as an “on–off” fluorescent probe for Cd^2+^ and l-Cys detection.

In this work, we first introduced 2-amino-3-hydroxypyridine and sodium borohydride as raw materials to fabricate boron and nitrogen co-doped carbon quantum dots (N,B-CQDs) with high fluorescence quantum yield (21.2%),which were synthesized through a simple, low-consumption and pollution-free one-pot hydrothermal method (Scheme 1). The obtained N,B-CQDs are able to detect Cd^2+^ and l-Cys rapidly and sensitively as an “on–off” fluorescent probe. When Cd^2+^ was added into the N,B-CQDs solution, the fluorescence of N,B-CQDs was sharply increased (turn on),which is attributed to the chelation enhanced fluorescence through the coordination reaction of surface functional groups with Cd^2+^. However, when l-Cys was introduced into N,B-CQDs/Cd^2+^ system, the fluorescence of the system was greatly quenched (turn off). This “on–off” fluorescence probe is selective and sensitive to Cd^2+^ and l-Cys with the detection limit of 0.45 µM and 0.28 µM, respectively. To our knowledge, this is the first paper to synthesize nitrogen–boron co-doped carbon quantum dots using 2-aminopyridine and sodium borohydride as N and B sources to detect Cd^2+^ based on fluorescence enhancement with “on–off” effect. Surprisingly, the probe is also suitable for Cd^2+^ and l-Cys analysis in real samples.

## 2. Experimental

### 2.1 Materials and characterization

Citric acid (99.5%), 2-amino-3-hydroxypyridine (97%) and CdCl_2_ (99%) were purchased from Macklin Biochemical Co., Ltd (Shanghai, China). l-Cysteine hydrochloride monohydrate (99%) was purchased from Aladdin Reagent Co., Ltd, China. Sodium borohydride (98%) was purchased from Aladdin Reagent Co., Ltd, China. 6 amino acids including l-Lysine (l-Lys, 98.5%), glycine (Gly, 99%), l-arginine (l-Arg, 98%), d-glutamic acid (d-Glu, 99%), l-histidine (l-His. 99%) and d-valine (d-Val, 98%) were purchased from Shanghai Xianding Biological Technology Co., Ltd (Shanghai, China). The metal salts used, including ZnSO_4_·7H_2_O (99%), KCl (99.5%), NaCl (99.5%), AgNO_3_ (99.8%), BaCl_2_·2H_2_O (99.5%), MgCl_2_·6H_2_O (98%), MnSO_4_·H_2_O (99%), CaCl_2_ (98%), K_2_CrO_7_ (99.8%), CoCl_2_·6H_2_O (99%) and CrCl_3_·6H_2_O (99%) were of analytical reagent grade and were used as received. Ultrapure water from a Ultrapure Water System (Nanjing QuanKun Bio-technology Co., Ltd, China) was used throughout the whole experiments.

UV-Visible absorption spectrum were measured by using a A11665 UV-Vis spectrophotometer (Shimadzu, Japan). Fourier transform infrared spectroscopy (FTIR) spectrum were performed on a IRAffinity-1 spectrophotometer (Shimadzu, Japan). The fluorescence measurements were recorded using an LF-1804005 fluorescence spectrophotometer (Thermo Fisher Scientific, China). The slit width was set at 5 nm for both excitation and emission. Transmission electron microscopy (TEM) and high resolution transmission electron microscopy (HRTEM) images were performed on JEM-3200FS (JEOL, Japan). X-ray photoelectron spectroscopy (XPS) were measured by ESCALAB 250 XI (Thermo Fisher Scientific, China).

### 2.2 Synthesis of nitrogen and boron co-doped carbon quantum

The B,N-CQDs was synthesized by one step hydrothermal. In a typical synthesis, firstly, 0.84 g citric acid and 0.5 g 2-amino-3-hydroxypyridine were dissolved in 30 mL of ultrapure water. Secondly, 0.08 g sodium borohydride was added. Then, the mixture was transferred to a teflon-lined autoclave (40 mL) and heated at 180 °C for 150 min. After cooling to room temperature, the above aqueous solution was dialysed through dialysis bag (1000 Da) for 48 h to remove raw materials. After dialysis, the solution was filtered through 0.22 µm membrane filter to remove the precipitate and frozen drying. Finally, the purified N,B-CQDs were brown powder and prepared for subsequent use.

### 2.3 Quantum yield measurement

The quantum yield (QY) of N,B-CQDs was calculated with quinine sulfate (whose QY is 54% (ref. 27) when dissolved in 0.1 M H_2_SO_4_) as a reference. In this paper, quinine sulfate and N,B-CQDs solutions were prepared, and all of them had absorbance less than 0.05 at 350 nm (the maximum excitation wavelength of the standard). The QY of the N,B-CQDs was evaluated by the following equation:*φ*_x_Y = *φ*_st_Y(*I*_x_/*I*_st_)(*A*_st_/*A*_x_)(*η*_x_/*η*_st_)^2^

Specially, *φ* symbolizes the QY, *I* symbolizes the fluorescence emission peak area, *η* is the refractive index of the solvent, and *A* is the absorbance at the excitation wavelength. The subscript “st” refers to standard and “x" refers to the sample. In our case, *η*_x_ = 1 and *η*_st_ = 1.

### 2.4 Detection of Cd^2+^ and l-cysteine

The sensing system of Cd^2+^ were prepared by mixing 4800 µL of ultrapure water, 100 µL of N,B-CQDs (0.1 mg·mL^−1^) and 100 µL of Cd^2+^ with different concentrations. Kept the total volume at 4 mL and the final concentration of N,B-CQDs was 2.5 µg mL^−1^. After reacting at room temperature for 1 min, the fluorescence spectra were recorded at the excitation wavelength of 360 nm. The sensing system of l-Cys were prepared by mixing 4700 µL of ultrapure water, 100 µL of N,B-CQDs (0.1 mg mL^−1^), 100 µL of Cd^2+^ (250 µM) and 100 µL of l-Cys with different concentrations. Then kept the mixed solution react for 1 min. The fluorescence spectra of the system were recorded in the same way mentioned above.

### 2.5 Detection of Cd^2+^ and l-cysteine in real sample

To investigate the practicality of the system, the N,B-CQDs sensing system was applied to the detection of Cd^2+^ in tap water and lake water samples. The trap water was filtered through a 0.22 µm microporous membrane. The lake water was filtered through a 0.22 µm microporous membrane and centrifuged at 8000 rpm for 10 min. Then the different concentrations of Cd^2+^ were added to the obtained water samples for three parallel experiments. Moreover, the practical application of the l-Cys determination in human urine was carried out. The human urine sample was centrifuged at 8000 rpm for 10 min firstly, after that, various concentrations of l-Cys in human urine sample was added in the N,B-CQDs/Cd^2+^ system.

## 3. Results and discussion

### 3.1 Characterization of N,B-CQDs

**Fig. 1:**
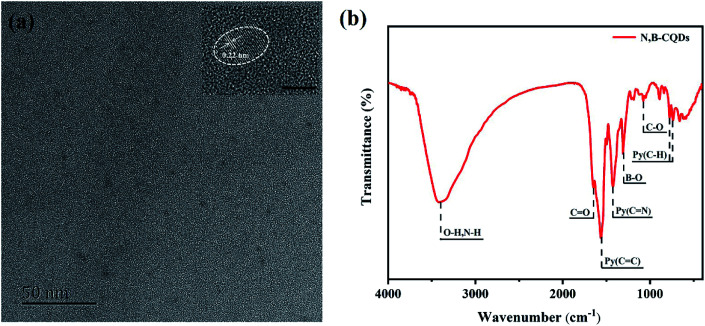
(a) TEM of N,B-CQDs (inset: HRTEM of N,B-CQDs, the bar: 5 nm). (b) FTIR of N,B-CQDs.

The TEM image (Fig. 1a) showed that N,B-CQDs were well monodispersed and the morphology of N,B-CQDs was spherical. Twenty particles were counted to evaluate the size distribution of N,B-CQD and the particle size was around 1.5–4 nm (Fig. S1†). The HRTEM image (inset in Fig. 1a) clearly showed that N,B-CQDs has a spherical structure, and the lattice spacing was 0.22 nm, which was related to the (100) lattice planes of graphite carbon.^28^

The surface groups and chemical bonds of N,B-CQDs can be explored by FTIR spectrum. As shown in Fig. 1b, the peak at 3400 cm^−1^ was indicated the stretching vibration of the O–H/N–H bond. The peak at 1640 cm^−1^ was designated as the stretching vibration of the C=O bond, which was due to the carboxyl in the surface of CQDs.^29^ The two peaks at 1561 cm^−1^ and 1425 cm^−1^ were related to the stretching vibrations of C=C and C=N bonds of aromatic skeleton. The peaks at 700–800 cm^−1^ were attributed to the bending vibration of aromatic ring hydrogen. All above proved that there was pyridine structure in the structure of N,B-CQDs.^30^ The peaks at 1302 cm^−1^ ascribed to the stretching vibration of B–O bond,^31^ suggesting the existences of B element in the CQDs framework. And the peak at 1076 cm^−1^ was attributed to the C–O stretching vibration of C–O–C bond.

**Fig. 2:**
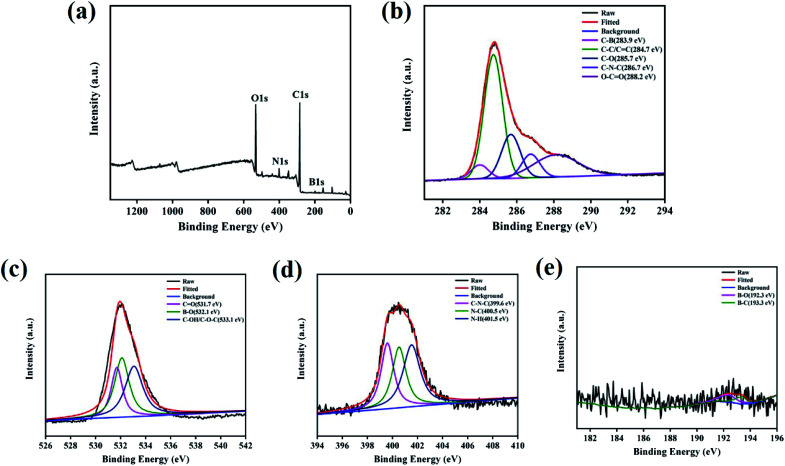
(a) XPS survey spectrum of N,B-CQDs. (b) C 1s, (c) O 1s, (d) N 1s and (e) B 1s XPS spectrum of N,B-CQDs.

To explore the elemental compositions of N,B-CQDs, the XPS spectra was investigated. The full scan of the XPS spectrum shown in Fig. 2a showed four different typical peaks at 285.9 eV, 532.5 eV, 401.3 eV and 199.1 eV attributed to carbon (C 1s), oxygen (O 1s), nitrogen (N 1s) and boron (B 1s) respectively, and their contents were 70.45%, 23.63%, 4.85% and 1.06%, respectively. The C 1s spectrum (Fig. 2b) was deconvoluted into five peaks at 283.9 eV, 284.7 eV, 285.7 eV, 286.7 eV and 288.2 eV, which were attributed to C–B, C–C/C=C, C–O, C–N–C and O–C=O. The O 1s spectrum (Fig. 2c) was deconvoluted into three peaks at 531.7 eV, 532.1 eV and 533.1 eV, which were owing to C=O, B–O and C–OH/C–O–C. The N 1s spectrum (Fig. 2d) was deconvoluted into three peaks at 399.6 eV, 400.5 eV and 401.5 eV, corresponding to C–N–C, N–C and N–H,^32^ respectively. The B 1s spectrum (Fig. 2e) was deconvoluted into two peaks with a peak at 192.3 eV for B–O and a peak at 193.3 eV for B–C.^33^ On the basis of the above results, both FTIR and XPS characterization methods have proved the successful doping of the element B and N.

### 3.2 Optical properties

**Fig. 3:**
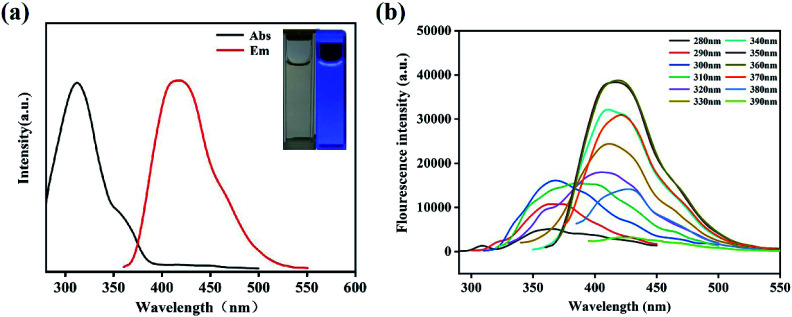
(a) UV-Vis absorption spectrum and fluorescence spectrums (inset: photographs of N,B-CQDs under the radiation of (left) visible light and (right) 365 nm UV light). (b) Fluorescence spectra of N,B-CQDs with different excitation wavelengths from 280 nm to 390 nm.

The N,B-CQDs solution was pellucid light yellow in natural light and displayed bluish violet under 365 nm UV light (inset in Fig. 3a). In order to further explore the optical properties of N,B-CQDs, N,B-CQDs was characterized by UV-vis absorption spectrum and fluorescence spectrum. As shown in Fig. 3a, there was an obviously absorption peak at 316 nm attributed to n–π* transition of the sp^2^ carbon, which were relevant to the oxygen-containing functional groups. The weak peak at 360 nm attributed to n–π* transition, which was indicated the aromatic π system with extended conjugation that possibly came from pyridine in the N,B-CQDs structures.^34^

To inquire into the fluorescent properties of N,B-CQDs, the photoluminescence (PL) emission behavior of the N,B-CQDs under different excitation wavelengths was investigated. As can be seen from the fluorescence spectrum (Fig. 3b), the fluorescence intensity presented a change law of first increasing and then decreasing with the excitation wavelength increasing from 280 nm to 360 nm. Moreover, the maximum photoluminescence peaks showed a significant red shift. This clearly shows the excitation-dependent PL behavior of N,B-CQDs, which was correspond to the properties of carbon quantum dots by previous publications. This excitation wavelength-dependent property may be caused by the optical selection of differently sized nanoparticles (quantum effect) or different emissive traps on the N,B-CQDs surface.^35,36^

With the photoluminescence quantum yield (PL QY) of quinine sulfate as the reference, the PL QY of N,B-CQD was about 21.2% at the excitation wavelength of 350 nm. This QY of the N,B-CQDs prepared in our work is higher than those previous researches reported,^37,38^ which illustrates the extraordinary fluorescent properties and good application prospect of the N,B-CQDs in our work. Then, the effects of temperature, UV light irradiation time, pH value, ionic strength on the fluorescence stability of N,B-CQDs were evaluated. It is clear that the fluorescence intensity of N,B-CQDs kept stable when the temperature increased from 10 to 50 °C (Fig. S2a†), which indicated that temperature had almost no effect on the character of N,B-CQDs. As shown in Fig. S2b,† the fluorescence intensity of N,B-CQDs had sharply changed with the pH changing from 2 to 11, which is due to the extensive protonation–deprotonation of the amide group of the N,B-CQDs.^39^ This suggests that the N,B-CQDs is pH responsive, which is differ from other CQDs reported by previous research.^40,41^ Furthermore, there was no obvious fluorescence intensity change of in the presence of UV light irradiation time (0–6 h) and different concentrations of NaCl (0.5–3 M) (Fig. S2c and d†). These results promised that the good photostability of N,B-CQDs and potential applications of N,B-CQDs under more severe conditions.

### 3.3 Detection of Cd^2+^

**Fig. 4:**
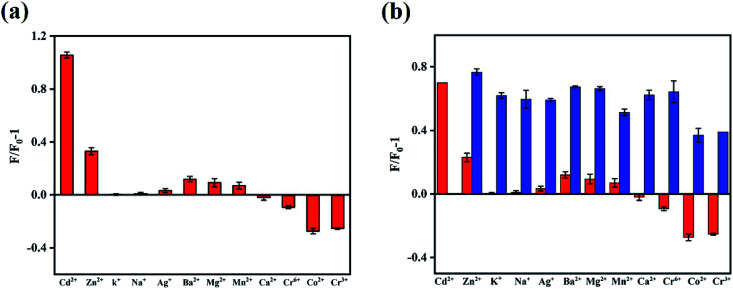
(a) Fluorescent response of N,B-CQDs to different metal ions (25 µM) in ultrapure water. *F*_0_ and *F* represent the fluorescent intensity of N,B-CQDs in the inexistence and existence of metal ions, respectively. (b) The fluorescent intensity of N,B-CQDs (red) and N,B-CQDs + Cd^2+^ (blue) solutions in the existence of various metal ions (25 µM). Excitation at 360 nm.

In order to obtain optimal reaction conditions, the sample pH value (a), testing medium (b) and incubation time (c) were optimized. As shown in Fig. S3,† the best experimental conditions were found when the Cd^2+^ detection was carried out in the pH = 7 ultrapure water after incubating with N,B-CQDs for 1 min. For the sake of the selectivity of the N,B-CQDs for Cd^2+^, the PL intensity variations before and after adding 250 µM of metal ions, including Cd^2+^, Zn^2+^, K^+^, Na^+^, Ag^+^, Ba^2+^, Mg^2+^, Mn^2+^, Ca^2+^, Cr^6+^, Co^2+^ and Cr^3+^ to the N,B-CQDs solution were investigated separately. As shown in Fig. 4a, the PL growth rate (expressed as *F*/*F*_0_ − 1) of N,B-CQDs is dramatically increased only in the presence of Cd^2+^,and other metal ions caused only little effect on the fluorescence intensity of the N,B-CQDs. Fig. 4b shows the anti-interference performance for N,B-CQDs detecting towards Cd^2+^. It is clearly indicated that there was no significant effect on the PL intensity of the N,B-CQDs in the presence of interference metal ions. However, when Cd^2+^ ions were added to the N,B-CQDs solutions, remarkable fluorescence enhancement was noticed. From these results we can infer the conclusion that the N,B-CQDs have an excellent selectivity in detection of Cd^2+^ ions.

**Fig. 5:**
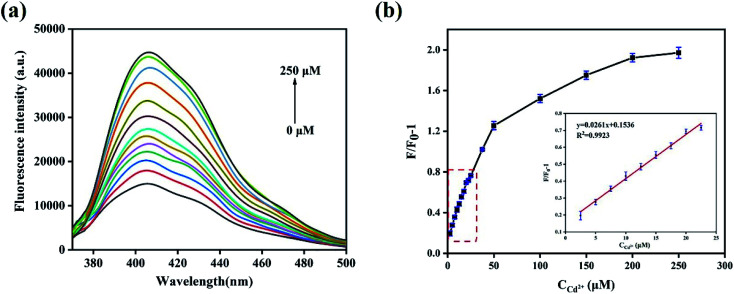
(a) Emission spectra of N,B-CQDs with various concentrations of Cd^2+^ in ultrapure water. (b) Relationship between fluorescence intensity variation and Cd^2+^ concentration ranging from 0 µM to 250 µM. *F*_0_ and *F* represent the fluorescent intensity of N,B-CQDs in the inexistence and existence of Cd^2+^ respectively (inset: the linear relationship between fluorescence intensity variation and Cd^2+^ concentration in the range from 2.5 µM to 22.5 µM). Excitation at 360 nm.

To evaluate the sensitivity of the N,B-CQDs for Cd^2+^, the detection limit for Cd^2+^ detection was explored. As shown in the Fig. 5a, the PL intensity was reduced gradually as the concentration of Cd^2+^ ions increased, implying that the PL intensity of the N,B-CQDs is very sensitive to Cd^2+^ ions. The Fig. 5b presents the relationship between the fluorescence intensity and the concentration of Cd^2+^. As illustrated in the inset of Fig. 5b, an excellent linear relationship with the concentration of Cd^2+^ in the range of 2.5–22.5 µM is observed. The linear equation was *F*/*F*_0_ − 1 = 0.1498 + 0.0263*C*_Cd^2+^_ with a good linear correlation (*R*^2^ = 0.9943). The limit of detection (LOD) for Cd^2+^ based on a signal-to-noise ratio of 3 was approximately 0.45 µM, which was comparable to other fluorescent probes based on carbon materials (Table 1). There are few studies about carbon quantum dots used to detect Cd^2+^, and this is the first paper to detect Cd^2+^ using N,B-CQDs based on fluorescence enhancement with “on–off” effect.

**Table 1:** Comparison of the proposed methods with previous methods employed for Cd^2+^ detection based on carbon materials

Methods	Linear range (µM)	Detection limit	Ref.
N,B-CQDs as fluorescence enhanced-probe	2.5–22.5	0.45 µM	This work
N,P-CDs as fluorescence enhanced-probe	0.5–12.5	0.16 µM	42
S,N-CDs using scallion (SL) as the carbon source	0.1–3.0, 5.0–30.0	15 nM	33

### 3.4 Possible mechanism of the detection for N,B-CQDs towards Cd^2+^

**Fig. 6:**
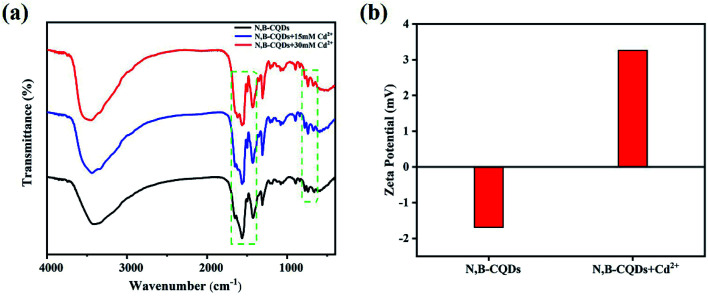
(a) FTIR spectra (b) zeta potential of N,B-CQDs without and with the addition of Cd^2+^ under the optimal experimental conditions.

Although carbon quantum dots fluorescence emission mechanism of the origin and mechanism of photoluminescence is unclear, it is widely accepted that there are two main types of fluorescence enhancement mechanism, the one is a metal surface enhanced fluorescence (MEF) or plasma enhanced fluorescence (SPEF),^43–45^ and another is a chelate enhanced fluorescence (CHEF), attributes to complex of the surface functional groups with metal ions.^46^ Studies have proposed that chelation between surface functional groups and metal ions can decrease internal charge transfer (ICT), improve photoinduced electron transfer, and enhance emission.^47,48^ In our work, the enhancement of fluorescence may be due to the decreased electron donor capacity of electron donor groups (amino and pyridine nitrogen) when N,B-CQDs chelate with Cd^2+^, thus quenching the ICT transition and improving the photoinduced electron transfer to N,B-CQDs/Cd^2+^ complex. As shown in the Fig. S4,† the absorption peaks of N,B-CQDs had no obvious change with the increase of Cd^2+^ concentration. This means the possibility of SPEF mechanism can be ruled out. In order to verify the formation of N,B-CQDs/Cd^2+^ complex, several kinds of characterization of the coordination of N,B-CQDs with Cd^2+^ or without Cd^2+^ were carried out. As illustrated in Fig. 6a, FTIR spectra indicated that the strength of the surface functional group of N,B-CQDs decreases gradually with increasing addition of Cd^2+^, and the new peak at 1612 cm^−1^ demonstrated the formation of N,B-CQDs/Cd^2+^ complex. Furthermore, The zeta-potential increased from −1.69 eV to 3.26 eV after the addition of Cd^2+^ (Fig. 6b), which was attributed to the electrostatic neutralization action. All the above results confirmed the chelation of N,B-CQDs with Cd^2+^.

### 3.5 Detection of l-cysteine using by N,B-CQDs/Cd^2+^ system

**Fig. 7:**
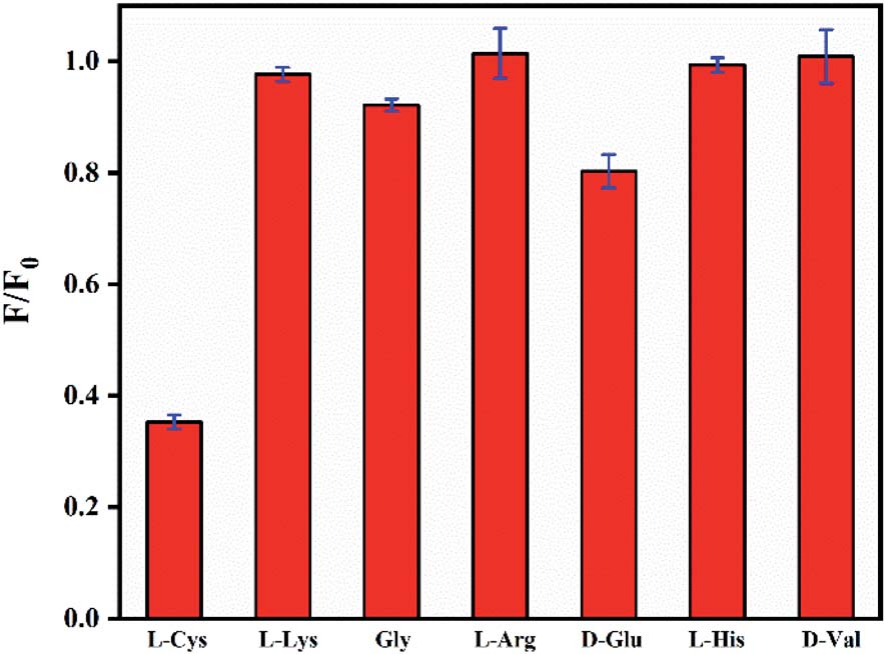
Fluorescent response of N,B-CQDs containing Cd^2+^ (250 µM) to amino acids (25 µM) in ultrapure water. *F*_0_ and *F* represent the fluorescent intensity of N,B-CQDs in the inexistence and existence of l-Cys respectively. Excitation at 360 nm.

**Fig. 8:**
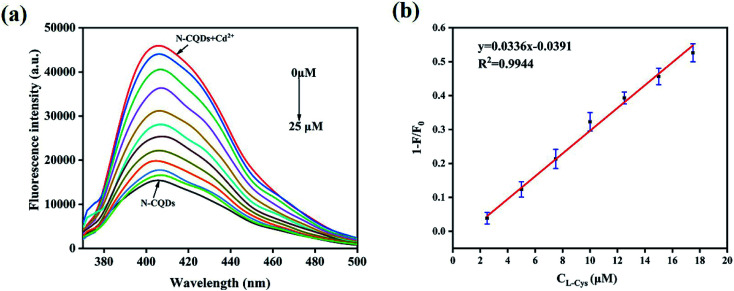
(a) Emission spectra of N,B-CQDs containing Cd^2+^ (250 µM) with various concentrations of l-Cys in ultrapure water. (b) The linear relationship between fluorescence intensity variation and l-Cys concentration in the range from 2.5 µM to 17.5 µM). *F*_0_ and *F* represent the fluorescent intensity of N,B-CQDs in the inexistence and existence of l-Cys respectively. Excitation at 360 nm.

Then, the fluorescence response of N,B-CQDs/Cd^2+^ system used for l-Cys detection was investigated in the presence of l-Cys. As shown in Fig. 7, the effects of six essential amino acids on the fluorescence signal of N,B-CQDs/Cd^2+^ system were investigated for comparison, only l-Cys exhibited notable quenching of the fluorescence. Thus, the system can be used for detection of l-Cys from different essential amino acids. As can be seen from Fig. 8a, with the concentration of l-Cys increasing, the fluorescence intensity of the system quenched gradually. This occurred because of that l-Cys have stronger binding preference toward Cd^2+^ than N,B-CQDs due to the formation of Cd^2+^–SR bond.^49^ The Fig. 8b presents the relationship between the fluorescence intensity and the concentration of l-Cys. There are a good linear relationship with the concentration of l-Cys in the range of 2.5–17.5 µM. And the linear equation was 1 − *F*/*F*_0_ = 0.0326*C*_l-Cys_ − 0.0329 with a good linear correlation (*R*^2^) of 0.9921. The LOD for l-Cys based on a signal-to-noise ratio of 3 was approximately 0.28 µM, which was lower than those previous work using by “on–off” fluorescent probes (Table 2). Therefore, N,B-CQDs have the advantages of simplicity, rapidity, good selectivity and high sensitivity when used as a “on–off” fluorescent probe for cysteine detection. Such detection method was comparable or superior to other traditional methods for the detection of l-Cys.

**Table 2:** Comparison of the proposed methods with previous methods employed for l-Cys detection using by “on–off” fluorescent probes

Methods	Linear range (µM)	Detection limit	Ref.
N,B-CQDs as fluorescent probes for “on–off”	2.5–17.5	0.28 µM	This work
Carbon dots as nanosensor by means of fluorescence “off–on” switching	2–20	0.29 µM	40
Nitrogen-doped carbon dots as fluorescent probes for “on–off”	0–56	97 nM	50
Nitrogen-doped carbon quantum dots as fluorescent probe for ‘‘off–on”	0–70	92.3 nM	51
Boron and nitrogen co-doped on–off–on carbon dots	5–175	2.3 µM	28

### 3.6 Application to real sample analysis

In order to evaluate the practicality of this fluorescent probe, the N,B-CQDs sensing system was applied to detecting Cd^2+^ in tap water and lake water samples. Cd^2+^ was not found in tap water and lake water samples. As listed in Table 3, the recoveries of Cd^2+^ in real samples are ranging from 97.9% to 102.2%, and the relative standard deviations (RSD) of three replicate detections for each sample below 5%. Moreover, the practical application of the l-Cys determination by N,B-CQDs/Cd^2+^ system in human urine was carried out. As shown in Table 4, the recoveries ranged from 101.8% to 105.9% with the relative standard deviations (RSD) of three replicate detections for each sample below 5%. These results indicated the reliability of our method for Cd^2+^ and l-Cys determination in real samples.

**Table 3:** Detection of Cd^2+^ in real samples (*n* = 3)

Sample	Add (µM)	Found (µM)	SD (µM)	Recovery (%)	RSD (%)
Tap water	5	4.92	0.05	97.90	1.2
	10	9.89	0.16	98.71	2.0
	15	15.3	0.41	101.8	3.3
Lake water	5	5.13	0.14	101.3	3.5
	10	10.2	0.27	102.2	3.3
	15	15.2	0.51	101.3	4.0

**Table 4:** Detection of l-Cys in real samples (*n* = 3)

Sample	Add (µM)	Found (µM)	SD (µM)	Recovery (%)	RSD (%)
Urine	5	5.12	0.16	101.8	0.9
	10	10.6	0.26	105.9	1.2
	15	15.6	0.23	104.0	1.5

## 4. Conclusion

In summary, we first introduced 2-amino-3-hydroxypyridine as nitrogen source to synthesize N,B-CQDs, and the quantum yield of N,B-CQDs reached up to 21.2%, which was higher than those in previous work. Based on the above characterization results, it can be seen directly that N atoms and B atoms were successfully doped into the structure of CQDs. N,B-CQDs was successfully used to detect Cd^2+^ and l-Cys in aqueous solution as a “on–off” fluorescent probe. Delightedly, it has proved that the fluorescence enhancement mechanism of N,B-CQD detecting Cd^2+^ is realized by a chelation enhanced fluorescence generated by the coordination reaction between surface functional groups and Cd^2+^. Simultaneously, a obviously fluorescence quenching was observed when l-Cys was added into N,B-CQDs/Cd^2+^ system, which is due to the formation of Cd^2+^–SR bond that frees up N,B-CQDs. In the two fluorescence “turn-on” and “turn-off” processes, this fluorescent probe obtained a good linear relationship over Cd^2+^ concentration ranging from 2.5 µM to 22.5 µM with a detection limit of 0.45 µM, while the concentration of l-cysteine showed a linear relationship in the range of 2.5–17.5 µM with a detection limit of 0.28 µM. The sensor has been successfully used to detect Cd^2+^ and l-cysteine in real samples with satisfactory results. Furthermore, due to the fact that the synthesis method of N,B-CQD was straightforward, rapid, time-saving and cost-effective, it has provided a possibility for the practical application of Cd^2+^ detection with high sensitivity and selectivity in the future.

## Conflicts of interest

There are no conflicts to declare.

## Supplementary Material

RA-012-D1RA08219A-s001
