# Supplementary material for: A highly selective and sensitive “on–off” fluorescent probe for detecting cadmium ions and l-cysteine based on nitrogen and boron co-doped carbon quantum dots
Source: RSC Adv. 2022 Mar 15;12(13):8202–10. doi: 10.1039/d1ra08219a (PMC8982326; doi:10.1039/d1ra08219a)
Supplement: RA-012-D1RA08219A-s001 [file RA-012-D1RA08219A-s001.pdf]

Electronic Supplementary Material (ESI) for RSC Advances.
This journal is © The Royal Society of Chemistry 2022

A Highly Selective and Sensitive "on-off" Fluorescent Probe for Detecting Cadmium Ions and L-cysteine Based on Nitrogen and Boron co-doped Carbon Quantum Dots

Zhihong Yan[1], Wei Yao[1], Kang Mai[3], Jiaqi Huang[1], Yating Wan[2], Huang liu[2*], Bo Cai[4*], Yi Liu[1,2,4*]

College of Pharmacy, Guangdong Pharmaceutical University, Guangzhou 510000, China
[2] School of pharmaceutical and chemical engineering, Guangdong Pharmaceutical University, Zhongshan 528400, China
[3] Zhongshan Carefor Daily Necessities Ltd, Zhongshan 528400, China
[4] Guangzhou OPSEVE Cosmetics Co. Ltd , Guangzhou 510000, China
[*] Corresponding author: E-mail: Liuyi915@126.com

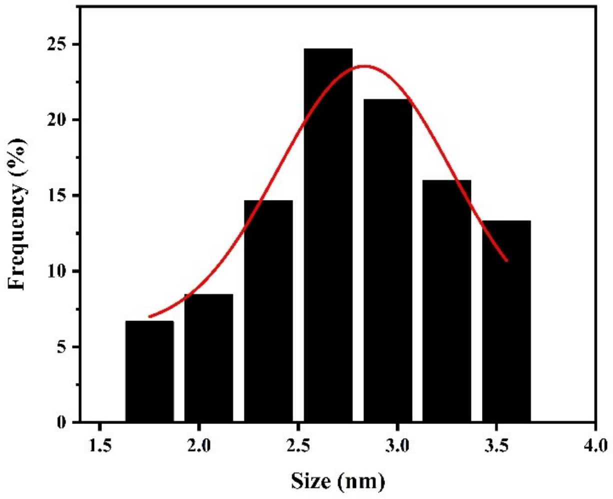

Fig S1. The histogram graph of size distribution of N,B-CQD.

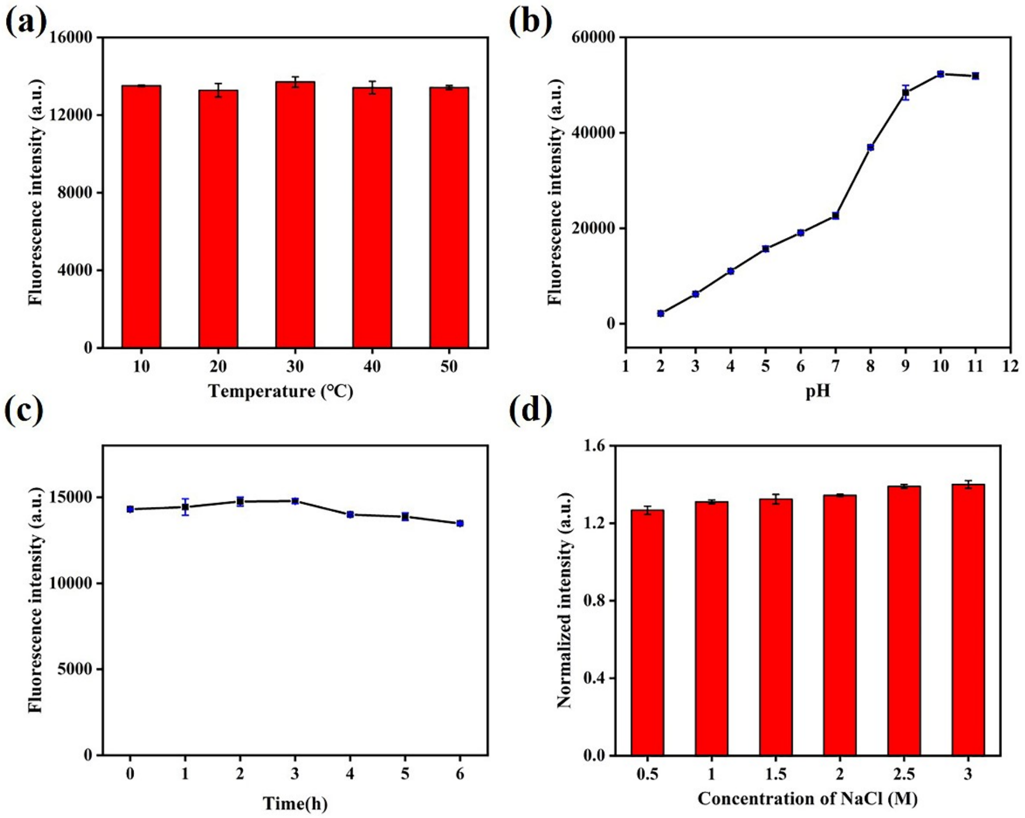

Fig S2. The effect of temperature **(a)**, pH **(b)**, UV light irradiation time **(c)** and ionic strength in NaCl aqueous solution **(d)** on the fluorescence intensity of N,B-CQDs. Excitation at 360 nm.

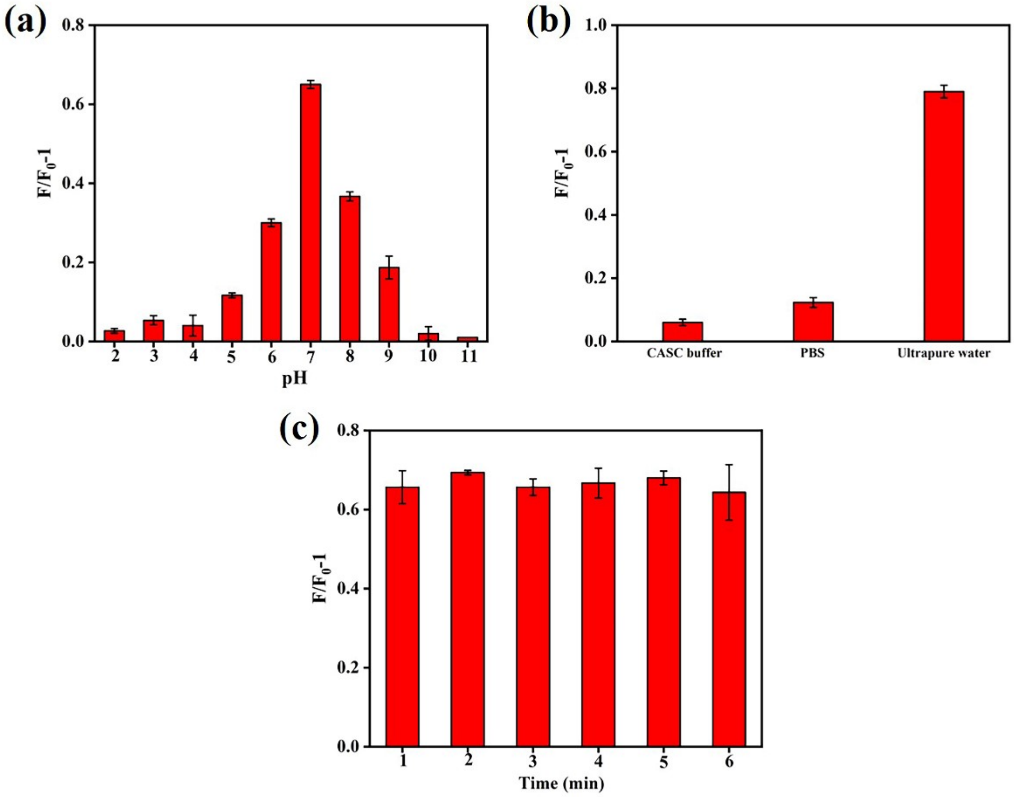

Fig S3. Effect of **(a)** sample pH value,**(b)** detection medium and **(c)** reaction time on the detection of Cd^{2+} with N,B-CQDs. Excitation at 360 nm.

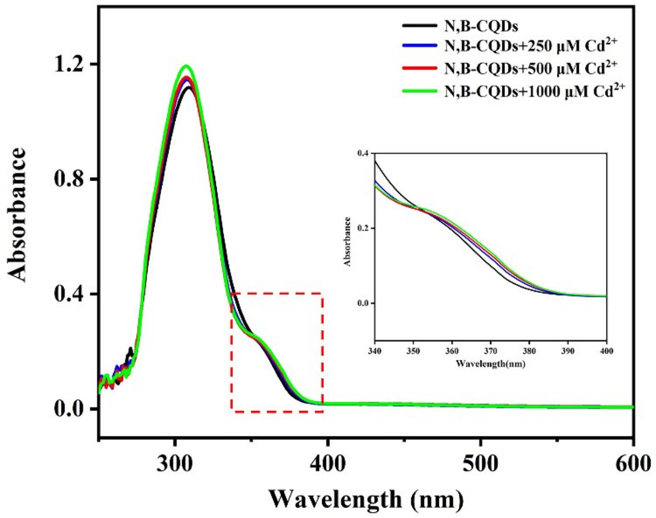

Fig S4. UV-Vis spectra for the N,B-CQDs with different concentrations of Cd^{2+} under the optimal experimental conditions.
